# What Have We Learnt About the Sourcing of Personal Protective Equipment During Pandemics? Leadership and Management in Healthcare Supply Chain Management: A Scoping Review

**DOI:** 10.3389/fpubh.2021.765501

**Published:** 2021-12-09

**Authors:** Stephanie Best, Sharon J. Williams

**Affiliations:** ^1^Australian Institute of Health Innovation, Macquarie University, Sydney, NSW, Australia; ^2^Murdoch Children's Research Institute, Melbourne, VIC, Australia; ^3^School of Health and Social Care, Swansea University, Swansea, United Kingdom

**Keywords:** supply chain management (SCM), leadership, pandemic, COVID-19, personal protective equipment (PPE), supply chain, resilience

## Abstract

**Introduction:** During the ongoing COVID-19 pandemic there have been much publicised shortages in Personal Protective Equipment for frontline health care workers, from masks to gowns. Recent previous airborne pandemics provide an opportunity to learn how to effectively lead and manage supply chains during crisis situations. Identifying and plotting this learning against time will reveal what has been learnt, when and, significantly, what can be learnt for the future.

**Aims:** (i) To identify the temporal trajectory of leadership and management learning in health supply chain management through pandemics and (ii) to identify leadership and management lessons to enable the resilient supply of key items such as PPE in future pandemics.

**Methods:** We undertook a scoping review in line with PRISMA (scoping review extension) searching Business Source Premier, Health Business Elite, Medline, ProQuest Business Collection and PubMed. Search terms were focused on recent airborne pandemics (SARS; Ebola; Zika virus; H1N1 swine flu, COVID-19), supply chain management, PPE, leadership, learning, inhibitors and facilitators and resilience e.g., SARS AND supply chain^*^ AND (“personal protective equipment” OR PPE) (leaders^*^ OR manage^*^) Titles and abstracts were downloaded to Endnote and duplicates removed. Two authors independently screened all of the titles and abstracts. Inclusion criteria focused on leadership and management in health supply chains during pandemics, peer reviewed or grey literature (either from business journals or reports): exclusion criteria included not in English and not focused on a named pandemic. Once interrater reliability was assured, authors completed a title and abstract screening independently. Ten percent of the resultant full text articles were screened by both authors, once agreement was reached the full text articles were screened independently noting reasons for exclusion. A data extraction tool was designed to capture findings from the final articles included in the review.

**Results/Discussion:** We found 92 articles and, after screening, included 30 full text articles. The majority were focused on COVID-19 (*N* = 27) and most were from the USA (*N* = 13). We identified four themes related to leadership and management of pandemic PPE supply chains, (i) *Leadership and management learning for pandemic PPE supply chain management*, (ii) *Inhibitors of PPE supply chain resilience during a pandemic*, (iii) *Facilitators employed to manage the immediate impacts of PPE supply chain demands during a pandemic*,and (iv) *Facilitators proposed to ensure longer term resilience of PPE supply chains during pandemics* Our study suggests there has been limited leadership and management learning for PPE supply chains from previous pandemics, however there has been extensive learning through the COVID-19 pandemic. Lessons included the importance of planning, the significance of collaboration and relationship building. Resilience of PPE supply chains was reported to be dependent on multiple levels from individuals to organisation level and also interdependent on (i) sustainability, (ii) the practise of PPE and (iii) long term environmental impact of PPE suggesting the need, long term, to move to a circular economy approach.

## Introduction

As a result of the Covid-19 pandemic there has been much attention internationally about the sourcing manufacture and supply of personal protective equipment (PPE) including surgical gowns, gloves and masks. Personal Protective Equipment (PPE), defined by OSHA19 as “specialised clothing or equipment worn by an employee to protect against infectious materials, which plays a key role in preventing the spread of infectious respiratory diseases (1–3) as well as the safety and well-being of healthcare workers and broader society in that it acts to break the chain of infection (4). PPE is a sector that has traditionally been dominated by a few global suppliers and due to the pandemic saw unprecedented demand along with the interruption of the manufacture and delivery of supplies (5). These supply issues are coupled with the lack of visibility of supply and the commonality of governments and healthcare procurement agencies buying through third parties which has further increased SC vulnerabilities (6). Such vulnerabilities have resulted in calls for greater transparency and understanding in terms of how PPE supply chains are managed and the factors that enable or inhibit their level of preparedness for critical events such as pandemics (5).

The management of supply chains relies on the active and systematic flow of goods and services, which includes all processes that transform raw materials into final products. One pre-COVID-19 US study reviews the lessons learned from the responses to the 2009 H1N1 influenza pandemic and the 2014 Ebola virus epidemic (2). It is evident that the PPE supply chain (manufacturing, distribution, and ordering) is complex, and a significant proportion of PPE goods is produced offshore and therefore likely to be slow to respond to any unexpected changes in demand. The upsurge in demand during the pandemic was not the only issue, the uncertainty relating to how long the response would last and how much produce would be needed also posed challenges in determining production and the increased capacity required. The importance of clear guidelines on the use of PPE has also been noted as a cause for concern, along with the need to coordinate supplies across regions so that stock can be moved quickly to where it is needed. Therefore, partnering with other facilities and suppliers was also critical.

COVID-19 pandemic was not the first outbreak to draw attention to the use and supply of PPE. Interest was heightened following the SARS outbreak of 2003 and the terrorist attacks around the 1990s and early 2000s (7). Despite the realisation of the importance of such equipment, the lessons learnt seem to have been limited (8) and possibly not reached those key decision makers in terms of how the PPE supply chains should be managed. This is evident by the fact that there is a lack of reporting regarding PPE and infection prevention and control protocols (9) in their work addressing the West African filovirus disease outbreak. The lack of standardisation for approval of use of PPE is also an issue (7), particularly in conditions like the ongoing pandemic where PPE may have to be shipped across countries, depending on supply and demand, where approval standards for using PPE could be different (10). These previous outbreaks have been an opportunity for learning in terms of how PPE supplies should be managed.

Despite having this knowledge of the PPE supply chain, most of these issues were witnessed again during the COVID-19 pandemic, especially during the early stages. Interruptions to supply resulted in the lack of PPE and an inequitable distribution (10). Global shortages of PPE were reported which were worsened due to the fact some items of PPE had to be worn not just by medical staff but the entire population. This surge in demand was exacerbated by panic buying and excessive stockpiling, which amplified the disruption in supply chains (11–13). The increased demand also resulted in an inflation in price due to the lower PPE stocks (10). Further problems in supply were due to the restrictions in travel, which saw countries having to start manufacturing and supplying their own PPE, which with the immediate need for the equipment raised questions regarding their approval for use according to existing standards which varied across countries (10). Understanding standards and product labelling is challenging, particularly as this is likely to vary between products. Frontline staff have likely used and been trained for particular brands and therefore introducing new equipment may require additional training or guidance.

As noted earlier most of the PPE used in Europe is produced offshore and there is a high reliance on a few global suppliers (14). Medical face masks, which are almost exclusively produced in China, have been in short supply during the COVID-19 pandemic in many industrialised countries which do not produce them (15). Strict lockdowns and other pandemic related restrictions imposed in supplier countries exacerbated this situation.

Critical disruptions in the PPE supply chain have also led to restrictions on the export of raw materials and supplies (13). Domestic shortages of PPE and the high uncertainty about future demand has led to governments and business leaders to be cautious. The shortages have also driven many domestic companies to rapidly reconfigure their supply chains (15). For example, the manufacture of facemasks in China rose from 20 million facemasks per day in January 14 to about 116 million per day at the end of February (16). In the UK, there were several organisations which repurposed their existing manufacturing facilities to make PPE (1, 17, 18). The reaction to the shortage of PPE in the UK has also come from perhaps some other unlikely sources. For example, the British fashion retailer Barbour is producing protective gowns (19). Burberry retooled its trench coat factory to non-surgical gowns and masks for patients (19). Louis Vuitton also announced they would be producing masks for front line workers along with their perfumeries such as Dior switching production to hydro-alcoholic gel hand sanitizer (20). Such innovations have enabled firms to extend their supply chains by creating separate channels of supply through the use of alternative and in some cases local providers or by restructuring international purchasing operations (15, 21). This rapid reconfiguration of supply chains has given rise to temporary supply chains, where rapid action must be taken in an uncertain and emergent environment (22). Suggestions have also been made that there is a need to use parallel supply chains for such critical items (4).

Although over the course of the pandemic the supply of PPE has stabilised, the initial problem was sufficient to highlight the lack of preparedness of almost all governments worldwide. For example, a recent review of the procurement and supply of PPE in England (4) during the early stages of pandemic showed that stock levels were dangerously low and required action from organisations outside of the sector to supplement supplies. The aim therefore of this scoping review is to (i) To identify the temporal trajectory of the learning for the leadership and management of PPE supply chains through pandemics and (ii) to identify leadership and management lessons to enable the continued supply of key items such as PPE in future pandemics.

## Methods

The use of scoping studies to synthesise research evidence is becoming increasingly popular (23). The field of supply chain management extends beyond academia and therefore a scoping review was undertaken in order to capture relevant grey literature. Factors to consider when undertaking a scoping review have been refined over the years (23, 24) though the same principle stages, identified by Arksey and O'Malley (25), remain in place: 1. Identifying the research question; 2. Identifying relevant studies; 3. Study selection; 4. Charting the data and; 5. Collating, summarising and reporting the results. We followed this structure to report our methods and findings.

### Stage 1. Identifying the Research Question

The aim and objectives for this study have been outlined in the introduction.

### Stage 2. Identifying Relevant Studies

We took a systematic approach to identifying articles relevant to the research aim following the PRISMA ScR extension (26). With the guidance of a specialist health management librarian, we identified five databases to interrogate: Business Source Premier, Health Business Elite, Medline, ProQuest Business Collection, and PubMed. Search terms related to virus disease infection pandemics, supply chains and leadership and/or management and resilience were established following discussions and trialling of test search terms. The final search ran as follows: [(SARS OR Ebola OR Zika virus OR H1N1 swine flu OR COVID-19 OR coronavirus) AND supply chain^*^ AND (“personal protective equipment” OR PPE) AND (leaders^*^ OR manage^*^)]. Duplicate references were discarded, and reference lists were mined to identify additional resources.

### Stage 3. Study Selection

Two reviewers (SB and SW) analysed all the title and abstracts independently applying inclusion and exclusion criteria (Table 1) using Rayyan (27) a web tool designed to promote expeditious collaborative systematic literature review. We included articles reporting empirical studies, reviews and commentary articles from peer reviewed studies and business reports. Studies not focused on the leadership and/or management of health PPE supply chains, including resilience, during a pandemic were not included. We were particularly interested in those publications recording lessons learnt and/or recommendations for managing PPE in future pandemics.

**Table 1:** Inclusion and exclusion criteria for articles.

**Inclusion**	**Exclusion**
Focus on supply chains, SCM and PPE and Pandemic and Leadership/Management	Focus on impact of lack of PPE or clinically focused
Empirical studies, reviews and commentaries/opinion pieces	No abstract
	Not in English
Grey literature: professional/business journals and reports	Grey literature: newspapers

Results from the independent title and abstract screening were reviewed by both members of the research team. Conflicts and articles coded as “maybe” were discussed and a resolution to either include or exclude was identified. The full texts of the remaining articles were assessed (SB and SW) for inclusion using the inclusion and exclusion criteria in Table 1.

#### Data Analysis

We used a conventional content analysis approach which is commonly employed with study designs aimed at describing a phenomenon (28). Here, the study intention is to respond to the research aim and objectives focusing on the phenomenon of supply chains during pandemics. Data related to leadership and/or management and barriers and/or enablers to supply chain resilience were extracted from the final selected articles.

## Results

### Stage 4. Charting the Data

**Figure 1:**
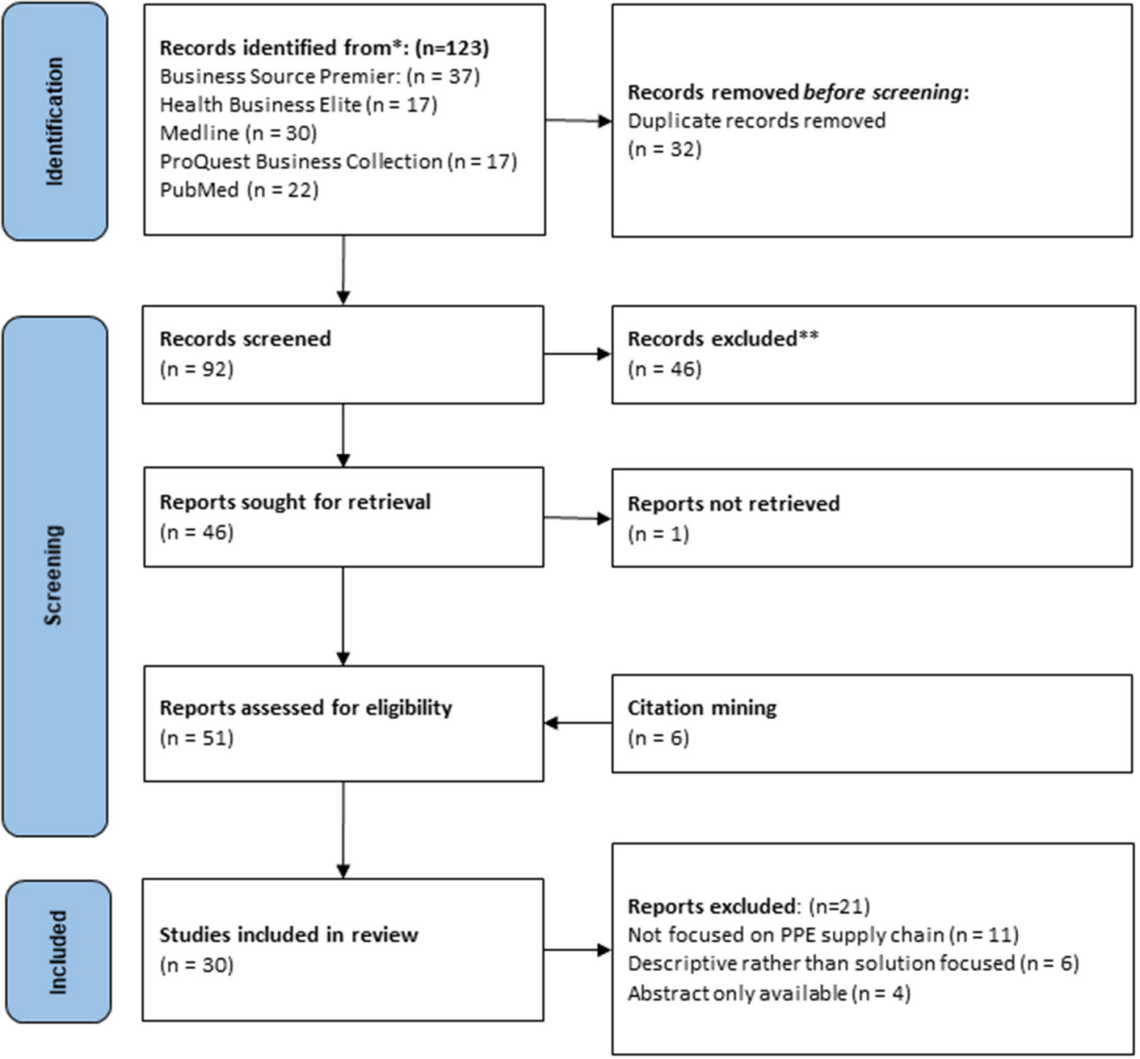
Details of screening process adapted from Page et al. (29).

Once duplicates were removed, we identified 92 articles for review. Title and abstract screening reduced this number to 45. One record was not retrieved, and four additional texts were added from citation mining and after full text screening we had a final selection of 30 articles for analysis. Figure 1 shows details including reasons for full text exclusions.

### Characteristics of Final Papers Selected

The majority of papers reported on PPE supply chains in relation to COVID-19 (*n* = 28) with only two reporting on either Ebola or H1N1. Of the 29 papers reviewed 27 were published since 2019 with only two being published between 2014 and 2017. Many papers were from the USA (*n* = 13) with two referring to global supply chain issues and several (*n* = 8) not reporting their location. Seventeen papers were peer reviewed and 13 sources were from the grey literature. A summary of the papers can be found in the table provided in the Supplementary Material.

### Stage 5. Collating, Summarising, and Reporting the Results

Data were extracted in relation to the leadership and management learning of PPE supply chains during a pandemic. Resilience was a common topic and so inhibitors and facilitators of resilience were also extracted. Here we present the findings by theme.

#### Leadership and Management Learning for Pandemic PPE Supply Chain Management

National health security is dependent on the medical supply chain, therefore leadership around the supply of PPE is critical (30, 31). Even prior to the COVID-19 pandemic we have seen global supply chains grow leaner and lacking to cope with disruptive challenges (30). It was noted that providers can only prepare as well as their leadership teams direct them (32). Leadership has been reported at various levels from international policy to individual team members. On the international stage high level leadership decisions impacted on the global PPE supply chain. China produces about half of the international supply of surgical masks and ramped up their production however they then stopped all mask exports (along with other countries such as Germany) leading to a global shortage (33). Decisions to stockpile exacerbated PPE shortages (33, 34) further compounded by unexpected consumers demand (35). However, organisational leadership was reported to be critical in setting the pace and tone of activity along the PPE supply and was reported to have been found lacking at critical decision points (32). The eventual lack of resources, PPEs and equipment exposed the healthcare supply chain fragility and dependency (5). Although failures in leadership were directed at stockpiling of PPE (32) others pointed to the under-preparedness of the PPE supply chain, the lack of policies (32, 33, 36, 37) seen in combination with a fragility of the system (38) and a lack of trust between stakeholders (33). There were calls to promote the transparency along the supply chain (36) to ensure both supplier and provider understand what is available at various stages of production and awaiting distribution (2).

The need for leaders to set up robust planning was a repeated theme with the need to plan centrally and implement locally identified by several authors (5, 30, 39) to improve transparency in the supply chain and contemporaneous access to PPE (30, 37). Additionally, there was the need to plan based on disaster type (i.e., how it spreads) and based on unique features of the type and usage of PPE (39), with some authors referring to how humanitarian supply chains are managed (5).

Collaboration and relationship building was reported as central to successful leadership and management of PPE supply chains (40–43). There is a need to ensure all parts of the supply chain are involved and engaged to promote the open innovation required to manage disrupted supply chains across organisational and national boundaries (33). There was a call for a reorientation of the practises of supply chain management with closer collaboration of suppliers and end users to develop projects in partnership with local industries (5). A particular challenge for the leadership of supply chain with the disruption caused by pandemics is that buyers rarely have the logistics knowledge and experience to manage a radically altered supply chain (42). Here building relationships has been central to enabling PPE supply chain pivot (38, 39).

Leadership at the team level was commonly reported to lead to benefits. For example, the team efforts required to meet the PPE supply chain demands led to a strengthening of interdepartmental relationships leading to greater interdependency within and between teams who could then rely on each other (40). Some leaders saw the demands of the pandemic as an opportunity to develop initiatives and partnerships they had been developing for some time and welcomed the opportunity to think beyond the traditional healthcare silos (38). Network formation and coordination was seen as important with three relationship types noted–new, established and established weak ties (44). This broadening of relationships supports diversity in the supply chain: previous pandemics have highlighted the need to encourage manufacturers to think expansively about sourcing for PPE and promote diversity (37, 40). New relationships extended not only to the sourcing and manufacturing of PPE but also to the usage of PPE (e.g., healthcare organisations working with local councils).

A novel experience was the impact of volunteers in the workplace as part of seeing people stepping up (40, 41). People taking up new roles outside the workplace, for example, people using 3D printers to make masks referred to as the “citizen supply chain” with patterns shared freely (45). Additionally, within the workplace cross training of staff was reported to increase flexibility and agility to respond (40, 42, 46). However, caution was expressed with a need to see these responses well-coordinated (41) to ensure user safety is not compromised (47).

Many authors provide suggestions for leaders to consider when looking to the future. Importantly there is a need to take time to reflect and then refine strategic plans (41, 48). There was a call for predictive tools that are bi-directional to anticipate demand though leaders and managers need to be able to trust the tool (32). The need for flexible and agile structures to be in place was reported in order to accommodate local and national PPE needs while ensuring visibility, resilience and responsiveness (30, 32, 49). Core components of agile PPE supply chains, to ensure responsiveness to pandemics, include flexibility, transparency, persistence and responsiveness, globally independent and equitable (36). During times of pandemics leaders who want to not only survive but also to thrive need to consider how they create value for their clients, prioritise breakthroughs over continuous improvement, collaborate, mitigate risk through shared funding and building resilience in local supply chains (41).

#### Inhibitors of PPE Supply Chain Resilience During a Pandemic

The leadership and management learning reported above present some of inhibitors to a resilient PPE supply chain. Additionally, there were reports of the overdependence on the few countries that have traditionally mass-produced PPE (33, 50). Despite experience from previous outbreaks, pandemic or other crisis (e.g., Hurricane Katerina) a failure to learn was identified, specifically the lack of cohesive strategy for PPE (2, 51). Previous supply chain practises including Lean, just-in-time (JIT) inventory and low unit of measure (LUM) were reported to have not promoted resilience to endure the surge demand of a pandemic (35, 52). Although the value of developing PPE supply chain resilience was recognised, there was acknowledgement that there was no quick fix to developing resilience, particularly while having to focus on immediate demands (35, 51). There has been a, perhaps understandable, unwillingness on behalf of manufacturers to invest the additional resources that would promote resilience and a need for commissioners to accept PPE may need to cost more (33). This will be challenging for some organisations who have experienced a loss of income from elective surgery leading knock-on effects on the supply chain (35) which has a knock-on effect on sustainability. Others point to the disorganisation of supply chains and bureaucratic inefficiencies that compromise preparedness for pandemic demands (33). Overcoming these barriers will require collaboration of key PPE suppliers, government reforms and reduction in “red tape” (47).

Schumacher et al. (14) suggest that the COVID-19 pandemic is an event that has exposed the varied global supply chain vulnerabilities and dependencies. Drawing on the work of Chopra (15) and Park et al. (13), they summarise these as being: stockpiling, capacity overload, rapid supply configuration, reduced or no visibility of the upstream supply chain; supply shortages, capacity loss in production and warehousing, *ad hoc* supply to end users without regular conformity checks and surging demand.

#### Facilitators Employed to Manage the Immediate Impacts of PPE Supply Chain Demands During a Pandemic

Lessons from previous pandemics point to the need for action at the community, clinician, day to day supply chain management levels. For example, at the community level, the Mayo clinic ran a “Lead by Example” campaign to encourage health care workers to promote the appropriate use of PPE when outside work (53) and so reduce unnecessary demand on the supply chain. The community also became directly involved in supply chain by making PPE, circumnavigating global supply chains. Availability of open-source designs for PPE, were freely shared to facilitate others to reproduce PPE e.g., face masks using 3D printing (45). This openness and transparency can lead to building long term relationships that could last beyond an immediate pandemic (38). For clinicians, there was a need to provide them with clear and consistent guidance on PPE usage to ensure they can stay safe while also preserving supplies (2, 53). Here, clear, timely and reliable communication was essential. Finally, at the day-to-day supply chain management level, improving the visibility of the demand and supply of PPE (2) and undertaking predictive surge modelling to promote transparency of available PPE supplies is needed to permit proactive planning (53).

#### Facilitators Proposed to Ensure Longer Term Resilience of PPE Supply Chains During Pandemics

A pandemic reinforces the significance of resilience and sustainability in supply chains and use of PPE, including the overuse of single use items (35) and several authors reported approaches to longer term sustainability of the supply of PPE, including the need to learn from previous pandemics (2, 32). Views on stockpiling varied with some in favour of early stockpiling (34) and others seeing this approach as hoarding (so reducing visibility of what stock is available) (36). However, ultimately health care supply chains need more diversification, localised systems and to move away from global supply chains in order to build more resilient communities (30, 32, 35, 52) with Handfield going further and noting core attributes for a supply chain fit to withstand a pandemic including flexibility, traceability and transparency, persistence and responsiveness and equitable access (30).

There was a call for innovation in conserving, obtaining and stock supplies (35). However, ensuring the production and delivery of safe and effective PPE is paramount. The lack of international standards for PPE was recognised (36) with calls to develop standard manufacturing protocols (e.g., for 3D printing for PPE) (45). To mitigate risk Schumacher promotes the need to be aware of what is happening up and down stream of the supply chain, setting up formal inspection processes where required (14).

## Discussion

Themes identified from this review related to leadership and resilience, (i) Leadership and management learning for pandemic PPE supply chain management, (ii) Inhibitors of PPE supply chain resilience during a pandemic, (iii) Facilitators employed to manage the immediate impacts of PPE supply chain demands during a pandemic, and (iv) Facilitators proposed to ensure longer term resilience of PPE supply chains during pandemics, describe the phenomenon of PPE supply chains during pandemics. First, the importance of leadership at all levels from government and individuals noting critical features such as planning, collaboration and relationship building. Second, barriers to resilience focused on historic PPE sourcing and traditional supply chain practises (e.g., JIT). Third, facilitators for resilience applied in practise included actions for multiple levels including, community, clinician and day to day supply chain management and stressed the importance of communication. Finally, some facilitators of resilience were proposed to support longer term benefit and diversification and delivery of supply chains closer to home.

One of our study aims was to report on the temporal trajectory of leadership and management learning for PPE supply chain management through pandemics. Surprisingly, only 2 of the final 29 articles reported on pandemics other than COVID-19 e.g., Ebola or H1N1. This is not to suggest there has been no learning from previous pandemics (54, 55), rather that the focus to date has not been on supply chains. Although several papers note the importance of learning lessons from previous pandemics [e.g., (56)] without a previous focus it is difficult to collate these into learning points for leaders and managers of PPE supply chains. The surge of papers centred on supply chains since COVID-19 may indicate previous pandemics did not experience any supply chain issues, or that there was a focus on other areas (e.g., clinical topics). The locations impacted by earlier outbreaks/pandemics (e.g., largely low-to-middle income countries) may not have attracted as much attention from academics, with exception of those studying humanitarian supply chains.

If we compare the key lessons that were recorded from Patel et al.'s (2) study of supply chain issues arising during the public health emergency response to Ebola with the more recent findings from our scoping review (Table 2). This would suggest that many of the issues were experienced during outbreaks prior to COVID-19 but not on the global scale recently seen. If we examine these issues many of them relate to those akin with supply chain vulnerabilities (5, 6, 13–15).

**Table 2:** Comparison of Supply chain issues identified from Pre and During the COVID-19 pandemic.

**Supply chain issues**	**Pre-COVID-19**	**During** **COVID-19**
Difficulties in predicting demand and supply	✓	✓
Overordering and stockpiling of PPE	✓	✓
Placing orders with multiple vendors	✓	✓
No-centralised order monitoring system	✓	✓
Reliance on a few global suppliers	✓	✓
Product use affected by regulatory requirement	✓	✓
Lack of SC strategies and policies		✓
Supply chain practises lacked resilience		✓
Reduced or no visibility of the upstream supply chain		✓

Future focus for leaders and managers in this field should include reviewing pandemic plans at national and local levels to establish whether learning from the COVID-19 pandemic, including diversifying supply chains and establishment of international standards for PPE has been instigated. Careful and ongoing monitoring of pandemic preparedness plans is clearly essential to avoid the disruption of the supply of PPE experienced globally through the COVID-19 pandemic. Decisions around being largely dependent on single global suppliers need to be reviewed considering recommendations made for at least dual sourcing and the use of parallel supply chains. This diversified supply chain could also be taken from a geographic perspective to limit the supply-side risk from one country, which includes the development of local suppliers. However, in the short-term there may well be a need to build robust inventories of PPE to buffer against other supply chain disruptions and longer term to consider regulation of PPE and the PPE supply chain to ensure standardisation (14, 33).

While examining resilience, several papers referred to sustainability. Even though the environmental impact of PPE was not the focus of this study it is clear that i) sustainability, ii) the practise of PPE i.e., discarding PPE easily, when not necessary, and iii) long term environmental impact of PPE are interconnected and difficult to disentangle. The circular economy proposes a move away from a linear business model where resources are consumed with a primary focus solely on the end goal (57). Instead, the circular economy seeks to breakdown functional silos, minimise waste, keep resources in use as far as is possible and enable end-to-end visibility, collaboration, and optimization (57, 58). Leadership lessons from this review feed into the development of a circular economy for PPE supply chain management. For example, the importance of communication where for instance clinicians promote conservation of PPE resources and reduce waste. Equally it is essential frontline staff are protected, therefore communication must be timely, accessible, up to date and accurate.

Following this, despite persistent shortage of PPE in some areas, there was a shift to understand the issues faced by people wearing PPE. Problems that came with the utilisation of PPE varied from, a need for more training regarding the donning and doffing (use) of PPE, lack of confidence regarding their use or an understanding regarding their level of protection provided, and adverse effect associated with use of PPE, especially over longer periods of utilisation (59, 60). And finally, at present with the tapering of the pandemic in some countries, attention is now being given to the disposal and environmental impact of PPE, which is not only related to the various ecosystems but also to infection control for those dealing with the PPE related waste management (61, 62).

If we again examine the temporal learning in terms of whether the lessons from earlier outbreaks are similar to those already emerging from literature concerning the COVID-19 pandemic. It would seem from comparing some of the key lessons from Ebola (2) and those presented from this review it would suggest that many of these were known (see Table 3).

**Table 3:** Comparison of key supply chain lessons identified from Pre and During the COVID-19 pandemic.

**Supply chain lessons**	**Pre-COVID-19**	**During** **COVID-19**
Tiered approach to categorise hospitals e.g., frontline facility, assessment hospitals, and treatment centres	✓	
Improve guidance: Include standards on products and guidance on how much of each product might be needed during a response	✓	✓
Monitor PPE use and distribution to minimise inappropriate purchases and improve overall distribution across the healthcare system.	✓	✓
Establish or centralise visibility on orders placed—need to reduce duplicate orders and understand true demand	✓	✓
Share supplies—Facilities within a community/regions should be encouraged to have plans in place to share products during an emergency	✓	✓
To have mechanisms in place to encourage the supply chain to have elasticity in the system to allow for increased supply in response to increases in demand need to be explored.	✓	✓
Investigate where to hold stock within the system in order to respond to increases in demand e.g., at the distributor, manufacturer or healthcare provider.	✓	
Improve domestic/local manufacturing surge capacity at the time of an event	✓	✓
Sharing of information and regular communication e.g., clear product specifications, demand information.		✓
Develop collaborative partnerships beyond the traditional healthcare silos	✓	✓
Increase visibility and transparency of supply chain practises		✓
Develop a framework for governance and response to enable a globally independent supply chain.		✓

### Limitations

We actively sought literature focused on the PPE supply chain during pandemics. Our search returned a limited number of papers relating to any pandemic other than COVID-19. Other broader literature from previous pandemics such as SARS or H1N1 may include some discussion about PPE supply chains and could be scoured to ensure no leadership or resilience lessons have been missed. Similarly, our selection of databases used for this review could have excluded some papers of interest.

### Conclusion

Sourcing Personal Protective Equipment (PPE) has been a global challenge for health systems during the COVID-19 pandemic. Such experiences are not new, for example Ebola, SARS. Yet global supply chains were still not sufficiently prepared or resilient to meet the demand. Learning and enacting lessons is essential to develop agile and sustainable solutions to ensure supply of appropriate and quality assured PPE in the event of future pandemics. The aims of this study were to focus on those lessons to be learnt by those involved in the leading and managing the sourcing of PPE and to identify the enablers and inhibitors to developing a continued and resilient source of supply, particularly during a pandemic.

Our review of the relevant literature suggests there has been limited leadership learning for PPE supply chains from previous pandemics, particularly around supply chain issues. However, there has been an increased attention on the PPE supply chain during the COVID-19 pandemic, which has led to the dissemination of the learning. Lessons included the importance of planning, transparency, the significance of collaboration and relationship building, which need to be in place long before the outbreak occurs (39). Healthcare systems are better prepared for pandemics or disasters when engaged in processes that can respond in a time of crisis. Arabi et al. (63) state the lessons learned from the COVID-19 pandemic should not just be contingency planning for times of “stress” but should reflect new habits that will strengthen the levels of preparedness for future events.

Resilience of PPE supply chains was reported to be dependent on multiple levels from individuals to organisation level and also interdependent on (i) sustainability, (ii) the practise of PPE and (iii) long term environmental impact of PPE suggesting the need to move to a circular economy approach. Such practises would enable supply chain tracking, tracing, and responsiveness supported by multiple levels of stakeholders—individual, organisational, supply chain, governmental, and community (57). However, Chopra (15) reminds us that there is a balance to be struck between efficiency and resilience, where organisations need to be prepared for a “shock” which is likely to be expensive, but at the same time the shock may never happen. He warns the biggest mistake is for manager so severely underestimate the probability of it happening.

Most studies in this review were based in higher income countries (such as the USA). As the global implications of COVID-19 become apparent it is evident that future research must include the leadership lessons from and for low- and middle-income countries to ensure the potential for an international response. Learning and enacting lessons is essential to develop agile and sustainable solutions to ensure a sustainable supply of appropriate and quality assured PPE in the event of future pandemics.

In this review we have limited our search to PPE and healthcare supply chains. However, it is evident that the challenges faced during the COVID-19 pandemic are similar to those when handling humanitarian issues in distributing relief items such as medicine and food as equitably as possible to the areas impacted by a disruptive event. Learning from other supply chain disruptions such as the Japanese tsunami could also provide some generic insights to supply chain vulnerabilities and resilience. Future research should therefore expand to draw lessons from learning from others industries how to mitigate supply chain disruptions [e.g., (64)] along with studying the responses seen from the humanitarian supply chain research [e.g., (65, 66)].

In 2006 Hick and Thorne reviewed the general lessons around the use, types and selection of PPE for emergency medical care and decontamination, they concluded their paper with the following statement: “*We can only hope that we are not forced to learn too many more harsh lessons about PPE use in the future. In the meantime, however, we should strive to prepare our communities by selecting appropriate protective technologies in relation to perceived threats and practising our responses so that our personnel are comfortable using their PPE and understand the consequences of not doing so”* [(7), p. 9]. The findings presented in this scoping review, suggests that more harsh lessons have been learnt from the disruptions during the COVID-19 pandemic particularly for those charged with leading and managing the supply of PPE. Corporate decision-makers when (re)designing supply chains need to “stress-test” for key performance measures such as resilience, responsiveness and reconfigurability. as well as focusing on traditional measures such as cost, quality, and delivery.

## Data Availability Statement

Articles informing this study are freely available. References are included in text and in the reference list. Requests to access the datasets should be directed to stephanie.best@mq.edu.au.

## Author Contributions

SB and SW co conceived the study, developed the study design, undertook data collection and analysis, and co wrote the first draught of the manuscript and the final submission. All authors contributed to the article and approved the submitted version.

## Conflict of Interest

The authors declare that the research was conducted in the absence of any commercial or financial relationships that could be construed as a potential conflict of interest.

## Publisher's Note

All claims expressed in this article are solely those of the authors and do not necessarily represent those of their affiliated organizations, or those of the publisher, the editors and the reviewers. Any product that may be evaluated in this article, or claim that may be made by its manufacturer, is not guaranteed or endorsed by the publisher.

## References

[1] Greenhalgh T Schmid MB Czypionka T Bassler D Gruer L. Face masks for the public during the covid-19 crisis. BMJ. (2020) 369:m1435. 10.1136/bmj.m1435 32273267

[2] Patel A D'Alessandro M Ireland K Burel W Wencil E Rasmussen S. Personal protective equipment supply chain: lessons learned from recent public health emergency responses. Health Secur. (2017) 15:244–52. 10.1089/hs.2016.0129 28636443

[3] Chu DK Akl EA Duda S Solo K Yaacoub S Schünemann HJ . Physical distancing, face masks, and eye protection to prevent person-to-person transmission of SARS-CoV-2 and COVID-19: a systematic review and meta-analysis. Lancet. (2020) 395:1973–87. 10.1016/j.jvs.2020.07.040 32497510 PMC7263814

[4] Seifert R. Digesting the Shocks: How Supply Chains Are Adapting to the COVID-19 Lockdowns (2020). Available online at: https://www.imd.org/research-knowledge/articles/supply-chains-adapting-tocovid-19/ (accessed August 18, 2021)

[5] Leite H Lindsay C Kumar M. COVID-19 outbreak: implications on healthcare operations. TQM J. (2020) 33:247–56. 10.1108/TQM-05-2020-0111

[6] Kwon I-W Kim S-H. Framework for successful supply chain implementation in healthcare area from provider's perspective. Asia Pac J Innov Entrep. (2018) 12:135–45. 10.1108/APJIE-04-2018-0024

[7] Hick J Thorne C. Personal protective equipment. Disaster Medicine. (2006) 2006:246–54. 10.1016/B978-0-323-03253-7.50043-1

[8] Sawada S Kuklane K Wakatsuki K Morikawa H. New development of research on personal protective equipment (PPE) for occupational safety and health. Indus Health. (2017) 55:8. 10.2486/indhealth.55-471 29212988 PMC5718767

[9] Hersi M Stevens A Quach P Hamel C Thavorn K Garritty C. (2015). Effectiveness of personal protective equipment for healthcare workers caring for patients with filovirus disease: a rapid review. PLoS ONE. 19:e0140290. 10.1371/journal.pone.0140290 26451847 PMC4599797

[10] Berklan JM. Analysis: PPE Costs Increase Over 1,000% During COVID-19 Crisis. McKnight's Long-Term Care News. (2020). Available online at: https://www.mcknights.com/news/analysis-ppe-costsincrease-over-1000-during-covid-19-crisis/ (accessed August 15, 2021).

[11] van Hoek R. Research opportunities for a more resilient post-COVID-19 supply chain–closing the gap between research findings and industry practice. Int J Oper Prod Manag. (2020) 40:341–55. 10.1108/IJOPM-03-2020-0165

[12] Ivanov D Dolgui A. Viability of intertwined supply networks: extending the supply chain resilience angles towards survivability. A position paper motivated by COVID-19 outbreak. Int J Prod Res. (2020) 58:2904–15. 10.1080/00207543.2020.1750727

[13] Park C-Y Kim K Roth S Beck S Kang JW . Global shortage of personal protective equipment amid COVID-19: supply chains, bottlenecks, policy implications. ADB Briefs. (2021) 130:1–10. 10.22617/BRF200128-2

[14] Schumacher R Glew R Tsolakis N Kumar M. Strategies to manage product recalls in the COVID-19 pandemic: an exploratory case study of PPE supply chains. Cont Resilience Rev. (2021) 3:64–78. 10.1108/CRR-07-2020-0024

[15] Chopra S. The Coronavirus Has Upended Supply Chains. Here's How Companies Can Prepare for the Next Disruption. (2020). Available online at: https://insight.kellogg.northwestern.edu/article/coronavirus-upended-supply-chains-how-companies-can-prepare-disruption (accessed August 21, 2021).

[16] OECD. The Face Mask Global Value Chain in the COVID-19 Outbreak: Evidence and Policy Lessons (2020). Available online at: https://read.oecd-ilibrary.org/view/?ref=132_132616-l4i0j8ci1q&title=The-Face-Mask-Global-Value-Chain-in-the-COVID-19-Outbreak-Evidence-and-Policy-Lessons&_ga=2.177689573.1518128158.1636536462-1568558115.1636536462

[17] Huddleston T. Tesla Engineers Are Building Ventilators for Coronavirus Patients Out of Car Parts—Take a Look. (2020). Available online at: https://www.cnbc.com/2020/04/06/video-tesla-buildingventilators-for-covid-19-patients-from-car-parts.html (accessed August 25, 2021).

[18] Shokrani A Loukaides E Elias E Lunt A. Exploration of alternative supply chains and distributed manufacturing in response to COVID-19; a case study of medical face shields. Mater Design. (2020) 192:108749. 10.1016/j.matdes.2020.108749 32341616 PMC7182752

[19] Wright G. Coronavirus: Burberry Offers Up Resources to Fight Against Outbreak. Retail Gazette. 30th March (2020). Available online at: https://www.retailgazette.co.uk/blog/2020/03/coronavirus-burberry-resources-outbreak-surgical-masks-donation-vaccine/ (accessed August 24, 2021).

[20] Nazir S. Louis Vuitton to Make Free Masks for Frontline Health Workers, Retail Gazette, 9th April. (2020). Available online at: https://www.retailgazette.co.uk/blog/2020/04/louis-vuitton-to-make-free-masks-for-frontline-health-workers/ (accessed August 24, 2021).

[21] Seifert R. Digesting the Shocks: How Supply Chains Are Adapting to the COVID-19 Lockdowns. (2020). Available online at: https://www.imd.org/research-knowledge/articles/supply-chains-adapting-tocovid-19/ (accessed August 18, 2021).

[22] Lundin R Soderholm A. A theory of the temporary organization. Scand J Manag. (1995) 11:437–55. 10.1016/0956-5221(95)00036-U

[23] Pham MT Rajić A. Greig JD Sargeant JM. Papadopoulosa A McEwen AA. A scoping review of scoping reviews: advancing the approach and enhancing the consistency. Res Synth Methods. (2014) 5:371–85. 10.1002/jrsm.1123 26052958 PMC4491356

[24] Levac D Colquhoun H O'Brien K. Scoping studies: advancing the methodology. Implement Sci. (2010) 5:69. 10.1186/1748-5908-5-69 20854677 PMC2954944

[25] Arksey H O'Malley L. Scoping studies: towards a methodological framework, Int J Soc Res Methodol. (2005) 8:19–32. 10.1080/1364557032000119616

[26] Tricco A Lillie E Zarin W O'Brien K Colquhorn H Levac D . PRISMA Extension for Scoping Reviews (PRISMA ScR): checklist and explanation. Ann Intern Med. (2018) 169:467–73. 10.7326/M18-0850 30178033

[27] Ouzzani M Hammady H Fedorowicz Z Elmagarmid A. Rayyan–a web and mobile app for systematic reviews. Syst Rev. (2016) 5:210. 10.1186/s13643-016-0384-4 27919275 PMC5139140

[28] Hsieh HF Shannon SE. Three approaches to qualitative content analysis. Qual Health Res. (2005) 15:1277–88. 10.1177/1049732305276687 16204405

[29] Page M McKenzie J Bossuyt P Boutron I Hoffmann T Mulrow C . The PRISMA 2020 statement: an updated guideline for reporting systematic reviews. BMJ. (2021) 372:n71. 10.1136/bmj.n71 33782057 PMC8005924

[30] Handfield R Finkenstadt D Schneller E Godfrey A Guinto P. A Commons for a supply chain in the post-COVID-19 era: the case for a reformed strategic national stockpile. Milbank Q. (2020) 98:1058–90. 10.1111/1468-0009.12485 33135814 PMC7772634

[31] NewsBeat. Help Keep Ebola Out of Human Supply Chains Material Handling & Logistics (2014). p. 8.

[32] Barlow RD. Saluting the resilience of COVID-19 Changemakers providers, suppliers push through pandemic-related logistics clogs and voids. Health Purchasing News. (2020), p. 12–21.

[33] Bhaskar S Tan J Bogers MLAM Minssen T Badaruddin H Israeli-Korn S . At the epicenter of COVID-19-the tragic failure of the global supply chain for medical supplies. Front Public Health. (2020) 8:562882. 10.3389/fpubh.2020.562882 33335876 PMC7737425

[34] Abedrabboh K Pilz M Al-Fagih Z Al-Fagih OS Nebel JC Al-Fagih L. Game theory to enhance stock management of Personal Protective Equipment (PPE) during the COVID-19 outbreak. PLoS ONE. (2021) 16:e0246110. 10.1371/journal.pone.0246110 33524057 PMC7850473

[35] Barlow R. What. Went Wrong. Health Purchasing News. (2020), p. 36–9.

[36] Finkenstadt DJ Handfield R. Blurry vision: Supply chain visibility for personal protective equipment during COVID-19. J Purch Supply Manag. (2021) 27:100689. 10.1016/j.pursup.2021.100689

[37] Haldane V Zhang Z Abbas RF Dodd W Lau LL Kidd MR . National primary care responses to COVID-19: a rapid review of the literature. BMJ Open. (2020) 10:e041622. 10.1136/bmjopen-2020-041622 33293398 PMC7725079

[38] Christ G. Healthcare, industry forge new supply chains in the fight against COVID-19. Modern Healthcare. (2020) 50. Available online at: https://www.modernhealthcare.com/supply-chain/healthcare-industry-forge-new-supply-chains-fight-against-covid-19

[39] Conway K. Lessons in disaster and demand planning. Heath Purchasing News. (2020), p. 54–5.

[40] Barlow R. Sustainability strives for attention, priority in pandemic-stricken world. Healthcare Purchasing News. (2020), p. 10–11.29787190

[41] Chuter D. Manufacturing leadership in the face of uncertainty. Manufacturers Monthly. (2020), p. 10–11.

[42] Smith E. Steering supplies on a steady track to needed destinations. Healthcare Purchasing News. (2020), p. 42–5.

[43] Conway K. Demand planning and forecasting: healthcare's time has come. Heath Purchasing News. (2020), p, 54.

[44] Vinson AH Fishstrom AB Rooney DM. Learning and collaboration during crisis: a novel university-community partnership to manufacture medical personal protective equipment. Int J Environ Res Public Health. (2021) 18:1–10. 10.3390/ijerph18052258 33668790 PMC7956350

[45] Belhouideg S. Impact of 3D printed medical equipment on the management of the Covid19 pandemic. Int J Health Plan Manag. (2020) 35:1014–22. 10.1002/hpm.3009 32567722 PMC7361600

[46] Meadmore S Turner L. How to manage a supply chain in a crisis. Electronics Weekly. (2020), p. 28–9.

[47] Singh SK Khawale RP Chen H Zhang H Rai R. Personal protective equipments (PPEs) for COVID-19: a product lifecycle perspective. Int J Prod Res. (2021). 10.1080/00207543.2021.1915511

[48] Francis J. COVID-19: implications for supply chain management. Front Health Serv Manag. (2020) 37:33–8. 10.1097/HAP.0000000000000092 32842087

[49] Handfield R Finkenstadt DJ Guinto P. How Business Leaders Can Prepare for the Next Health Crisis. Boston, MA: Harvard Business Publishing (2021).

[50] Dey S Cheng Q Tan J. All for one and one for all: why a pandemic preparedness league of nations? Health Policy Technol. (2020) 9:179–84. 10.1016/j.hlpt.2020.04.009 32427167 PMC7227598

[51] Atkinson CL McCue C Prier E Atkinson AM. Supply chain manipulation, misrepresentation, and magical thinking during the COVID-19 pandemic. Am Rev Public Adm. (2020) 50:628–34. 10.1177/0275074020942055

[52] Sharma A Gupta P Jha R. (2020). COVID-19: Impact on health supply chain and lessons to be learnt. J Health Manag. 22:248–61. 10.1177/0972063420935653

[53] Zorn CK Pascual JM Bosch W Thiel DD Francis D Casler JD . Addressing the challenge of COVID-19: one health care site's leadership response to the pandemic. Mayo Clin Proc Innov Qual Outcomes. (2021) 5:151–60. 10.1016/j.mayocpiqo.2020.11.001 33521584 PMC7833323

[54] Lautenbach E Saint S Henderson D Harris A. Initial response of health care institutions to emergence of H1N1 Influenza: experiences, obstacles, and perceived future needs. Clin Infect Dis. (2010) 50:523–7. 10.1086/650169 20064038 PMC2811228

[55] Rebmann T Wagner W. Infection preventionists' experience during the first months of the 2009 Novel H1N1 influenza A pandemic. Am J Infect Control. (2009) 37:e5–16. 10.1016/j.ajic.2009.09.003 20004810 PMC7132719

[56] Elston J Cartwright C Ndumbi P Wright J. The health impact of the 2014-15 Ebola outbreak. Public Health. (2017) 143:60–70. 10.1016/j.puhe.2016.10.020 28159028

[57] Nandi S Sarkis J Hervani A Helms M. Redesigning supply chains using blockchain-enabled circular economy and COVID-19 Experiences. Sustain Prod Consumpt. (2021) 27:10–22. 10.1016/j.spc.2020.10.019 33102671 PMC7566799

[58] Murray A Skene K Haynes K. The circular economy: an interdisciplinary exploration of the concept and application in a global context. J Bus Ethics. (2017) 140:369–80. 10.1007/s10551-015-2693-2

[59] Davey S Lee B Robbins T Randeva H Thake C. Heat stress and PPE during COVID-19: impact on healthcare workers' performance, safety and well[1]being in NHS settings. J Hosp Infect. (2021) 108:185–8. 10.1016/j.jhin.2020.11.027 33301841 PMC7720696

[60] Tabah A Ramanan M Laupland KB Buetti N Cortegiani A Mellinghoff J . Personal protective equipment and intensive care unit healthcare worker safety in the COVID-19 era (PPE-SAFE): an international survey. J Crit Care. (2020) 59:70–5. 10.1016/j.jcrc.2020.06.005 32570052 PMC7293450

[61] Ammendolia J Saturno J Brooks A Jacobs S Jambeck J. An emerging source of plastic pollution: Environmental presence of plastic personal protective equipment (PPE) debris related to COVID[1]19 in a metropolitan city. Environ Pollut. (2021) 269:116160. 10.1016/j.envpol.2020.116160 33316501 PMC7833877

[62] Kumar H Azad A Gupta A Sharma J Bherwani H Labhsetwar N . COVID-19 creating another problem? Sustainable solution for PPE disposal through LCA approach. Environ Dev Sustain. (2021) 23:9418–32. 10.1007/s10668-020-01033-0 33071605 PMC7546389

[63] Arabi YM Azoulay E Al-Dorzi HM Phua J Salluh J Binnie A . How the COVID-19 pandemic will change the future of critical care. Intensive Care Med. (2021) 47:282–91. 10.1007/s00134-021-06352-y 33616696 PMC7898492

[64] Chopra S Sodhi M. Reducing the Risk of Supply Chain Disruptions, MIT Sloan Management Review, 18 March. (2014). Available online at: https://sloanreview.mit.edu/article/reducing-the-risk-of-supply-chain-disruptions/ (accessed August 18, 2021).

[65] Malmir B Zobel C. An applied approach to multi-criteria humanitarian supply chain planning for pandemic response. J Human Logist Supply Chain Manag. (2021) 11:320–46. 10.1108/JHLSCM-08-2020-0064

[66] Dubey R Bryde D Foropon C Tiwari M Dwivedi Y Schiffling S. An investigation of information alignment and collaboration as complements to supply chain agility in humanitarian supply chain. Int J Prod Res. (2021) 59:1586–605. 10.1080/00207543.2020.1865583

[67] Wright G. Barbour Uses Supply Chain to Make PPE for Frontline Healthcare Workers, Retail Gazette, 16th April. (2020). Available online at: https://www.retailgazette.co.uk/blog/2020/04/barbour-supplies-ppe-nhs-frontline-healthcare-workers/ (accessed August 24, 2021).

