# Supplementary material for: What Have We Learnt About the Sourcing of Personal Protective Equipment During Pandemics? Leadership and Management in Healthcare Supply Chain Management: A Scoping Review
Source: Front Public Health. 2021 Dec 9;9:765501. doi: 10.3389/fpubh.2021.765501 (PMC8695796; doi:10.3389/fpubh.2021.765501)
Supplement: Supplementary file 1 [file Table_1.pdf]

	Author	Year	Title	Country	Grey/ Peer	Pandemic	Personal and Protective Equipment where referred to specifically
1	Abedrabboh	2021	Game theory to enhance stock management of Personal Protective Equipment (PPE) during the COVID-19 outbreak	UK	Peer	COVID-19	Not specified
2	Atkinson	2020	Supply Chain Manipulation, Misrepresentation, and Magical Thinking During the COVID-19 Pandemic	USA	Peer	COVID-19	Not specified
3	Barlow	(November) 2020	Sustainability strives for attention, priority in pandemic-stricken world	Not stated - ?USA	Grey	COVID-19	Refers to gowns, bouffant caps, skull caps, fabric masks and shoe covers.
4	Barlow	(August) 2020	What. Went. Wrong? Supply Chain struggles with its early response to explosive coronavirus pandemic	Not stated - ?USA	Grey	COVID-19	Refers to personal protective equipment (PPE) and disinfecting/sanitizing products
5	Barlow	(July) 2020	Saluting the resilience of COVID-19 Changemakers Providers, suppliers push through pandemic-related logistics clogs and voids	Not stated - ?USA	Grey	COVID-19	Refers to gloves, masks, respirators and sanitisers
6	Belhouideg	2020	Impact of 3D printed medical equipment on the management of the Covid19 pandemic	USA	Peer	COVID-19	3D printed masks
7	Bhaskar	2020	At the Epicenter of COVID-19–the Tragic Failure of the Global Supply Chain for Medical Supplies	China	Grey	COVID-19	Refers to PPE including N95 masks and surgical masks
8	Christ	2020	Healthcare, industry forge new supply chains in the fight against COVID-19	USA	Grey	COVID-19	Refers to face masks, respirators, gloves and gowns
9	Chuter	2020	Manufacturing leadership in the face of uncertainty	Australia	Grey	COVID-19	Not specified

**10**	Conway	(Oct) 2020	Lessons in disaster and demand planning	USA	Grey	COVID-19	Not specified
**11**	Conway	(Sept) 2020	Demand planning and forecasting: Healthcare's time has come	USA	Grey	COVID-19	Not specified
**12**	Dey	2020	All for one and one for all: Why a pandemic preparedness league of nations?	Not stated	Peer	COVID-19	N95 respirators, surgical gowns, gloves and masks, 'to name a few'
**13**	Francis	2020	COVID-19: Implications for Supply Chain Management	USA	Peer	COVID-19	Not specified
**14**	Haldane	2020	National primary care responses to COVID-19: a rapid review of the literature	Global	Peer	COVID-19	Not specified
**15**	Handfield	2020	A Commons for a Supply Chain in the Post-COVID-19 Era: The Case for a Reformed Strategic National Stockpile	USA	Peer	COVID-19	Not specified
**16**	Handfield	2021	How Business Leaders Can Prepare for the Next Health Crisis	USA	Grey	COVID-19	Not specified
**17**	Leite	2020	COVID-19 outbreak: implications on healthcare operations	Not stated	Peer	COVID-19	Refers to PPE generally with one mention of gowns
**18**	Malmir	2021	An applied approach to multi criteria humanitarian supply chain planning for pandemic response	Global	Peer	COVID-19	Includes face masks and face shields, and hand sanitiser
**19**	Meadmore	2020	How to manage a supply chain in a crisis	Not stated	Grey	COVID-19	Not specified
**20**	News Beat	2014	Help Keep Ebola Out of Human Supply Chains	West Africa	Grey	Ebola	Not specified

**21**	Schumacher	2020	Strategies to manage product recalls in the COVID-19 pandemic: an exploratory case study of PPE supply chains	UK	Peer	COVID-19	Refers to PPE generally with one mention of medical masks
**22**	Smith	2020	Steering supplies on a steady track to needed destinations	USA	Grey	COVID-19	Refers to PPE generally with one mention of masks
**23**	Vinson	2021	Learning and Collaboration during Crisis: A Novel University-Community Partnership to Manufacture Medical Personal Protective Equipment	USA	Peer	COVID-19	N95 masks, face shields and PPE in general
**24**	Zorn	2021	Addressing the Challenge of COVID-19: One Health Care Site's Leadership Response to the Pandemic	USA	Peer	COVID-19	Not specified
**25**	Finkenstadt	2021	Blurry vision: Supply chain visibility for personal protective equipment during COVID-19	USA	Peer	COVID-19	PPE in general with mentions of masks, gowns and gloves
**26**	Singh	2021	Personal protective equipment (PPEs) for COVID-19: a product lifecycle perspective	Not specified	Peer	COVID-19	"*Examples of PPE include such items as gloves, foot and eye protection, protective hearing devices (earplugs, muffs) hard hats, respirators, and full bodysuits*"
**27**	Patel	2017	Personal Protective Equipment Supply Chain: Lessons Learned From Recent Public Health Emergency Responses	USA	Peer	H1N1 and Ebola	PPE in general with mention of N95 face masks, surgical masks
**28**	Park	2021	Global shortage of personal protective equipment amid COVID-19: supply chains, bottlenecks, and policy implications	UK	Peer	COVID-19	Includes protective suits and surgical masks, gowns, and gloves

**29**	Chopra	2020	The coronavirus has upended supply chains. Here's how companies can prepare for the next disruption	Not specified	Grey	COVID-19	Mentions hand sanitizer and masks
**30**	Sharma	2020	COVID-19: Impact on Health Supply Chain and Lessons to be learnt,	India	Peer	COVID-19	Mentions face protection, goggles and mask or face shield, gloves, gown, head cover and rubber boots

Supplementary Material: Characteristics of papers selected for analysis
